# Where there’s smoke, there’s fire: insights from murine models on the effect of cigarette smoke in rheumatoid arthritis development

**DOI:** 10.3389/fimmu.2025.1588419

**Published:** 2025-05-20

**Authors:** Courtney Lynn Marshall, Mahadevappa Hemshekhar, Neeloffer Mookherjee, Liam J. O’Neil

**Affiliations:** ^1^ Department of Immunology, University of Manitoba, Winnipeg, MB, Canada; ^2^ Manitoba Centre for Proteomics and Systems Biology, Department of Internal Medicine, University of Manitoba, Winnipeg, MB, Canada

**Keywords:** rheumatoid arthritis, collagen-induced arthritis, cigarette smoke, risk factors ra, cs, rf, rheumatoid Factor, PTMs

## Abstract

Rheumatoid arthritis (RA) is a chronic autoimmune inflammatory disease characterized by joint inflammation and damage, leading to disability and pain. The etiology of RA is undefined but considered multifactorial, as interactions between genetics and environmental factors lead to the generation of autoantibodies that target synovial joints. Smoking is a well-established and widely studied risk factor for RA development and is associated with a reduced response to treatments and poor clinical outcomes. Murine models of inflammatory arthritis have provided many insights into the pathogenesis of RA and have recently been used to explore the relationship between cigarette smoking and RA. In this review, we comprehensively appraise the current literature investigating cigarette smoke exposure in murine models of inflammatory arthritis, focused on RA. The current literature indicates that the influence of smoke exposure on molecular and disease outcomes depends on the timepoint of exposure and genetic background of the mice. Further, dose-dependent increases in disease manifestations reproduce human clinical data that the intensity of smoking is linked to disease but demosntrate that there may be a plateau effect. Finally, we consolidate mechanistic findings to describe a potential mechanism through which cigarette smoke exacerbates murine arthritis. Understanding how these factors, genetics, timing, and intensity of exposure modulate response to CS in inflammatory arthritis models may lead to better drug development and personalized treatment strategies, ultimately improving outcomes for RA patients with a smoking history.

## Background

Rheumatoid arthritis (RA) affects over 17 million people worldwide a number that is expected to increase to 31 million by 2050 (1). The disease is characterized by chronic joint synovitis or inflammation, which if left untreated, results in bone erosion and cartilage destruction, leading to progressive joint damage and functional disability. Although the precise etiology of RA is not fully defined, it is well established that the disease develops over several stages spanning many years referred to as the pre-clinical stage of RA. During this phase, typically occuring several years prior to arthritis onset, genetic and environmental risk factors interact, leading to a loss of self-tolerance and the development of autoantibodies, such as rheumatoid factors (RF) and anti-citrullinated protein antibodies (ACPA) (2, 3). ACPA targets citrullinated antigens, this occurs when peptidylarginine deiminase (PAD) enzymes, which largely originate from neutrophils, convert arginines amino acids to citrullines. PAD enzymes and citrullinated antigen are increased in the joint, serving as a pool of autoantigen and contribute to the overall vicious inflammatory cycle seen in RA (4, 5).

There is a strong genetic component of RA and this is demonstrated by the heritability of the disease, which is as high as 60% (6). The HLA-DRB1*04 (DR4) allele is widely recognized as a significant risk factor for RA (3), specifically ACPA+ disease. DRB1 risk alleles, otherwise known as the shared epitope (SE), are involved in the production of the Major Histocompatibility Complex (MHC) II, which is a protein that is directly involved in the presentation of antigens by specialized cells, typically referred to as antigen presenting cells (APC). It has been shown that citrullinated peptide-MHC affinity is greatly enhanced by the presence of SE, which likely predisposes an individual to RA-specific autoimmunity. In addition, DQA1*0301/DQB1*0302 (DQ8) occurs in linkage with DR4 and has a similar enhanced affinity for presenting citrullinated antigens (3).

Of all the modifiable exposures associated with RA, the risk imparted by cigarette smoke (CS) is one of the most extensively described in humans. Numerous epidemiological studies have shown that smoking increases the risk of RA onset [previously reviewed in (7)]. Interestingly, there is also a clear interaction between smoking and SE whereby the combination of the two amplifies the risk of RA, compared to individuals exposed to only one of these risk factors (8). There is also evidence that smoking influences the generation of ACPA, potentially through enhanced citrullination in the lungs, leading to loss of tolerance (9–11). In addition to increased risk development, CS exposure is linked to increased severity of RA and a reduced response to therapeutics, suggesting it can play a dual role in increasing the burden of RA morbidity by increasing the prevalence and severity of the disease (12, 13). Although the associations between smoking and RA risk are widely accepted, the precise mechanism by which CS enhances RA development and progression remains unclear. Understanding how CS modulates RA will improve our understanding of the etiology of RA and may help optimize treatment strategies for RA patients who have or currently smoke.

Murine models have been instrumental in uncovering immunological and molecular mechanisms driving the pathogenesis of RA, including the identification of novel therapeutic targets [ie. TNF, IL-6R, Janus Kinase (14)] which has significantly altered patient outcomes. These murine models of inflammatory arthritis replicate many features of RA such as synovial hyperplasia, immune cell infiltration, cartilage degradation and bone erosion (15, 16). In addition, shared immunopathological features include the generation of auto-antibodies, and immune cell and cytokine profiles. As such, murine models of inflammatory arthritis have been used to explore the impact of CS on various aspects of RA, including development, severity, and the modulation of immunological mechanisms. Due to the differences in methods and timing of induction of arthritis, each model has distinct advantages and disadvantages, particularly in the context of CS exposure (15, 16). To our knowledge, the collagen-induced arthritis (CIA) and antigen-induced arthritis (AIA) models are the only models that have been used to study the effects of CS on murine arthritis. The CIA model is the gold standard for arthritis research, whereby sensitization with type II collagen (CII) in Freund’s adjuvant in DBA/1 mice induces autoimmune arthritis that appears ~28 days, with significant mouse to mouse variability, but shares multiple features with RA (17). The CIA model has been modified to include an injection of LPS on day 25 which increases incidence to 100% and disease onset occurs within 24-48h, also synchronizing disease in mice (18, 19). However, to our knowledge, the LPS synchronized CIA model has not been used to explore the effects of CS. The CIA model requires the development of both an adaptive and innate immune response resulting in both systemic and articular inflammation. Notably, the disease is driven by the production of pathogenic autoantibodies [anti-CII, ACPA, and RF, reviewed in (15)]. Genetic manipulation of CIA, using DR4 and DQ8 human transgenic mice, have also been investigated for their contribution to murine arthritis using a modified protocol (20). Importantly, transgenic mice carrying HLA-DR4 or HLA-DQ8 have increased susceptibility to develop arthritis after only one administration of CII, and have a propensity to also develop ACPA and RF (3, 20), perhaps more closely mimicking human RA. The AIA model is induced with an intra-articular (i.a) injection of methylated bovine serum albumin (mBSA) after three sensitization injections (day 0,7, and 14) in C57Bl/6 mice (15). Although this model shares many joint destructive features of RA, AIA leads to a monoarticular arthritis, which does not recapitulate the polyarticular disease that is observed in human RA and murine CIA (21). In this review, we summarize the effect of CS on CIA and AIA and the potential factors contributing to disparity in results, identify potential mechanisms, and make suggestions for future studies.

## How does exposure to CS modulate experimental arthritis?

### Inhalation of CS in CIA and AIA models

Murine arthritis CS models have generally exposed mice via i. conventional inhalation or ii. collection of particulate matter as “cigarette smoke condensate” and administering this via injection or intranasally. Most commonly, inhalation strategies are used and these studies are summarized in **Table 1** (22–26). Mice are typically exposed to CS in controlled chambers whereby smoke is directed from lit cigarettes into the chamber to be inhaled by mice. This approach aims to recapitulate real-world exposure to CS and is similar to how humans inhale CS. The amount of cigarette smoke exposure has varied within and between studies. Lindblad et al. was the first study to carry out this type of exposure model which combined an emphysema and CIA model. Mice were exposed to CS 16 weeks *before* the first CII administration followed by another 6 weeks of CS exposure after for a total of 22 weeks of exposure, with a CII booster on day 21 (26). In this study, extended pre-exposure to CS, perhaps counterintuitively, delayed the onset of clinical arthritis, reduced levels of anti-CII antibodies, and reduced the number of aCCP-positive animals (26). Clinical arthritis scores and joint destruction were similar between groups at the termination of the experiment (26). Using CIA others have exposed mice *after* CII sensitization; for example Kang et al. exposed CIA mice after one CII administration to 4 weeks of CS (22); Talbot et al. and Donate et al. exposed AIA and CIA mice to CS on day 12 and 17. For AIA mice this occurred throughout the mBSA sensitization period (days 0, 7, 14) but before the intra-articular mBSA challenge, for CIA mice this was 12 and 17 days after the first and only CII administration (23, 24); Heluany et al. exposed AIA mice to 1 week of CS starting on day 14 (Between the third mBSA sensitization and i.a. challenge) (25). Irrespective of the exposure protocol, these studies consistently showed that CS exposure following sensitization exacerbated arthritis severity (22–25). In the CIA model, CS exposure increased arthritis severity score and incidence, while demonstrating earlier onset of disease and more rapid accumulation or arthritis than unexposed mice (22, 23). A similar finding was shown in the AIA model, where CS exposure led to increased joint edema and mechanical hyperalgesia, indicating greater joint pain (23–25). The effects of CS were not limited to the joint; systemically, CS exposure led to increased circulating citrullinated proteins, such as vimentin and enolase, but, interestingly, not collagen II (22). Exposure to CS also enhanced circulating nicotine, cotinine (the main metabolite of nicotine), and heavy metal levels were seen in animals exposed to cigarette smoke (25). One study demonstrated increase in the level of serum ACPA in CS exposed CIA mice, although to our knowledge this has not been replicated elsewhere (22, 23). Within the joint, multiple studies have shown that CS increased joint destruction and immune cell infiltration into the joint, specifically neutrophils and neutrophil extracellular trap (NETs) formation in synovial fluid (22–25). CS also appears to alter the Th17-neutrophil axis, as demonstrated by increased Th17 cells and *Il17a* expression, which are known mediators of RA in humans (23–25, 27). The above data demonstrates that CS exposure after sensitization with collagen or antigen exacerbates disease in CIA and AIA models. This reproduces clinical data that CS exposure enhances risk of developing RA through rapid increase in incidence and increases severity of disease as demonstrated by increased scores, joint damage, and immune cell infiltration in joint. However, the contrasting impact on arthritis severity based on the timing of CS exposure relative to the sensitization phase in a murine model requires further exploration.

**Table 1:** Selected studies showing cigarette smoke (CS) modulates experimental arthritis.

First author	Model of Arthritis	Cigarette smoke exposure	Outcomes
Lindblad et al. (26)	CIA in DBA1/J (Male)	Exposure to 4 cigarettes, 6 days a week 16 weeks prior to CII adminsitration and continued for 6 weeks after.	• CS delayed onset of arthritis • CS did not alter histology of joint, but less animals affected in CS group • Serum IL-6 was unchanged • CS lowered level of IgG against CII • Lower number of aCCP-positive animals in CS group
Kang et al. (22)	CIA in DBA1/J (Female)	Exposure 1h per day at high dose (600ug/L) or low dose (150ug/L), 5 days a week for 4 weeks starting 1 day after CII administration.	• CS exposure dose dependently increased arthritis score and incidence • CS exposure dose dependently increased inflammation score of joint • CS exposure dose dependently increased destruction score of joint (cellular infiltration, pannus formation, cartilage damage) • CS exposure dose dependently increased citrullinated proteins in tarsal joints (vimentin) • CS exposure increased citrullination in trachea and bronchioles • CS exposure enhanced citrullinated vimentin, enolase, and filaggrin in serum. • CS exposure enhanced ACPA/a-CCP
Talbot et al. (23)	AIA in C57BL/6 (Male) CIA in DBA1/J male mice	Exposure to 1, 2,or 3 cigarettes per day, on days 12 and 17 after first mBSA sensitization. Exposure to 2 cigarettes per day, on days 12 and 17 after CII administration	• CS exposure dose dependently increased mechanical hyperalgesia • CS exposure dose dependently increased in neutrophil infiltration in joints. • CS exposure dose dependently increased *il17a* mRNA and Th17 cells in DLNs • CS exposure increased incidence and arthritis score • CS exposure increased neutrophils in joint.
Donate et al. (24)	AIA in C57BL/6 (Male)	Exposure to 2 cigarettes per day on days 12 and 17 after first mBSA sensitization.	• CS exposure enhanced neutrophilia in joint • CS exposure enhanced mechanical hyperalgesia • CS exposure enhanced histopathological score
Heluany et al. (25)	AIA model C57BL/6 (Male)	Exposure for 1h twice a day from days 14 to 20 after first sensitization with mBSA.	• CS exposure enhanced Nicotine, Cotinine, and metals in serum • CS exposure enhanced mechanical hyperalgesia • CS exposure enhanced edema, • CS exposure enhanced NETs in synovial fluid • CS exposure enhanced MCP-1 in synovial fluid • CS exposure reduced DLN and Spleen cellularity • CS exposure enhanced splenocyte IL-10 and reduced IL-2 in response to PMA. • CS exposure enhanced ROS
Bidkar et al. (35)	Transgenic DR4 mice (Female)	Exposure to 2 cigarettes 10 times a day for 5 days a week, with antigen exposure in the final week.	• Cs exposure increased PAD4 and decreased PAD2 expression in lung • CS exposure decreased T cell proliferation in response to citVIM peptide • CS did not enhance any antibody responses • CS did not alter T reg
Vassallo et al. (33)	Transgenic DQ8 and DR4 mice (Female and Male)	Exposure 3h a day for 5 days a week for 2 weeks before CII adminsitration and continued up to 10 weeks after. CS/CII were exposed to CS for 2 weeks then sensitized to CII and CS exposure continued for another 2 weeks. CII/CS mice were sensitized with CII then exposed to CS for 2 weeks.	• CS did not alter severity or incidence in male DR4 mice but lowered incidence and severity in female DR4 mice • CS enhanced incidence and severity in DQ8 mice without sex differences • CS enhanced Anti-CII antibodies but decreased anti-cit rVim and didn’t change RF in DR4 mice. • CS enhanced RF in DQ8 mice but did not change anti-CII or cit-CII cit-r-VIM antibodies. • CS enhanced LN responses to cit CII but decreased response to cit-r-VIM in DR4 mice. • CS enhanced LN responses to citCII in DQ8 mice • CS enhanced IL-17, IL-13, IL-10, TSLP, PAD2 and PAD4 expression in lung in DQ8 mice. • CS suppressed IL-17, IL-13, IL-19, PAD2 and PAD4 expression in lung of DR4 mice. • LNCS from CS DR4 mice increased IFNy, TNFa, IL-2, IL-13, IL-9, RANTES, GM-CSF, and MIP1a in response to CII • LNCs from CS DQ8 mice increased IFNy, TNFa, IL-10, and MIP1a in response to CII • DQ8 CII/CS enhanced LNC responses to native and cit CII and rVim • DR4 CS/CII mice increased LNC responses to native and cit CII.
Lin et al. (34)	Transgenic DQ8 mice	Exposed for 45 minutes, 10 times a day/5 days a week/2 weeks prior to CII sensitization (day 1) and continued until day 32.	• CS exposure enhanced incidence and severity of arthritis • CS exposure did not enhance anti-CII or ACPA

CIA, collagen-induced arthritis; AIA, Antigen-induced arthritis; CII, Type II Collagen; CS, Cigarette Smoke; LNC, Lymph Node Cells; r-Vim, recombinant Vimentin; Cit-CII, Citrullinated CII; cit-R-Vim, Citrullinated recombinant vimentin; aCCP, anti-cyclic citrullinated protein; ACPA, anti-citrullinated protein antibodies; RF, Rheumatoid Factor.

### Impact of CS concentrate on CIA

In contrast to inhalation exposure, others have used cigarette smoke condensate (CSC), whereby the particulate matter from mainstream and side stream smoke are collected and administered via injection or intranasally. In the CIA model, administration of CSC with the CII/CFA administration was associated with aggravated arthritis as represented by increased incidence and mean arthritis score (28, 29). Intraperitoneal administration one day before sensitization with CII had similar effects on arthritis severity. Injecting CSC bypasses the interactions of CS with the lung, which has been hypothesized to be an early site of inflammation and autoantibody formation in RA (11). Hence, Okamoto et al. administered varying concentrations of mainstream CSC intranasally seven and one days before CII adminsitration (30). Although two exposures prior to CII adminstration enhanced incidence and severity of CIA, a single exposure 24h prior to CII administration was enough to enhance incidence and arthritis scores (30). Although findings of CSC exposure align with those of CS inhalation exposure in that CS enhances CIA, it is important to consider how exposure to CSC differs from inhalation of CS. Comparing studies of traditional CS exposure via inhalation to CSC is difficult, most studies fail to indicate how CSC content corresponds to cigarette smoke exposure, Chujo et al. suggested that 100μg of CSC corresponds to exposure of 32 cigarettes (28). Further, which compounds enter the bloodstream from CS inhalation compared to injection are not entirely clear. If CSC injection continues to be used, a deeper analysis of circulating compounds with a comparison to inhalational CS may help determine optimal injection schedules and key differences of these 2 exposure systems. Overall, the administration of CSC further adds to data supporting that CS enhances incidence and severity of arthritis.

## What are the factors that may influence CS-mediated modulation of murine arthritis?

Studies have varied widely in the genetics of the mice and CS exposure protocol. By comparing the results of the various studies, we identified that the genetics of the mice, timing and intesntiy of CS exposure exert modulatory effects on CIA. These are factors are also currently under investigation for human RA as well (31). As such, the identified variables require consideration when interpreting studies and designing future experiments.

### Do genes matter? the use of transgenic animals to study cigarette smoke and CIA

Human studies have identified important gene-environment interactions between smoking and genetic risk alleles which appear to synergize to enhance RA risk, specifically seropositive disease (8, 32). Thus, transgenic models of DQ8 and DR4, which recapitulate the shared epitope genetic risk allele, provide a valuable tool to investigate this interaction (3, 20). Vassallo et al. (33) exposed DR4 and DQ8 mice for 2 weeks prior to CII sensitization and continued for 10 weeks in the CIA model. Mice carrying the DR4 allele were largely unaffected or experienced a lower incidence compared to filtered air (FA) exposed mice. Contrarily, CS-exposed DQ8 mice experienced greater incidence and severity compared to FA-exposed mice. CS also enhanced incidence and severity of CIA, and anti-CII antibody production in DQ8 mice in a study reported by Lin et al. (34). However, as this study only employed DQ8 mice, we cannot compare altered susceptibility to non genetically modified mice or DR4mice. Additionally, response to CS varied with allelic susceptibility, for example, DQ8 mice had increased production of RF (along with enhanced severity). CS also enhanced co-stimulatory molecule expression of lung dendritic cells and Th2 and Th17 gene expression in the lungs of DQ8 mice but not DR4 mice. In all, these data demonstrate that genetic background modulates response to CS, suggesting that DQ8, rather than DR4 risk allele impart higher risk of severe arthritis in CIA. Another study by Bidkar et al. (35) explored the differential modulation of immune responses between HLA DRB1*0401 mice (arthritis susceptible) and *0402 (resistant to arthritis) and CS. Although they did not report the incidence and severity of CIA, CS exposure resulted in greater expression of PADs in the lungs of *0401 mice, which may influence the production of RA specific antigens for ACPA generation (7). T cell responses to vimentin and cit-vimentin also trended to increased IFNγ and IL-23 production in *0401 mice, but IL-10 in 0402 mice, indicating greater systemic inflammation in *0401 mice. Consistent with this, *0402 mice displayed a higher proportion of Treg cells in response to CS, which are immunosuppresive. Although these data clearly demonstrate a gene-environment ineraction, it remans unclear why DR4 mice appear to be non-susceptible to modulation by CS exposure (33). Further understanding of the mechanism that different MHC alleles confer risk and how it is altered by CS is an area that warrants exploration.

### Does timing and genetic of CS matter?

As described above, there appears to be a substantial differential effect of CS exposure on murine arthritis depending on the timing of the exposure, either before CII sensitization (delaying onset of arthritis by at least 3 days and reducinga autoantibody production) or after (accelerates disease onset and pathogenesis) (22–26). Interestingly, manipulation of the genetic background of the mouse appears to influence the relationship between timing of exposure and arthritis severity. This was robustly demonstrated by Vassallo et al (33), who sensitized DR4 and DQ8 mice to CII with two distinct protocols varied by when CS exposure occurred, either before or after CII sensitization. The first group, (CII/CS) was sensitized to CII and then exposed to CS for 2 weeks. The other group (CS/CII) was exposed to CS for 2 weeks then sensitized with CII followed by another 2 weeks of CS exposure. Following each protocol, they anayzed lymph node cell (LNC) responses to native and citrullinated CII and r-Vim. Both DR4 and DQ8 mice themselves responded differently in the same protocol, DQ8 mice showed increased LNC responses in the CII/CS protocol compared to DR4 mice. In the CS/CII protocol, DR4 mice seemingly generated greater resposnes compared to DQ8. When comparing protocols within the same genetic group, there were clear differences in LNC responses based on protocol. This demonstrates that the timing of exposure will alter immune responses which is dependant on genetic background. The timing of CS exposure has recently gained interest in the clinical field, and the stages of CIA and AIA that potentially mirror human RA development can be leveraged to uncover when CS exposure exerts its greatest impacts on RA development including in auto-antibody generation (36). A recent study determined when throughout the stages of development of RA genetics and smoking play a role and found that smoking is involved in autoantibody (ACPA) and symptom generation but may not drive progression of symptoms (31). By leveraging the timing and genetic modification of mice in CIA and AIA models, we can confirm these observations and identify when smoking confers the greatest risk in different individuals based on genetics.

### Does the amount of CS matter?

Several studies have employed different intensities of exposure in parallel allowing us to determine if amount of cigarette smoke is a key factor in modulating experimental arthritis. This is important because in human RA, it has been demonstrated that there is a dose-response to cigarette exposure in RA risk. For example, the impact of smoking less than 10 pack-years compared to over 20 pack-years inicreases the risk of RA development from 26% to 94% (37). Overall, these studies primarily show that CS exposure has a dose-dependent increase in disease aggravation (22, 23). Using traditional CS exposure in chambers, Kang et al. compared low and high concentrations (150ug/L to 600ug/L) while Talbot compared exposure of 1, 2, or 3 cigarettes (22, 23). Kang et al. showed dose-dependent effects on citrullination in the joints, trachea, and serum, as well as ACPA positivity, and that higher concentrations accelerated incidence and arthritis score (22). Similarly, Talbot et al. found increased mechanical hyperalgesia, *il17a* expression in DLNs, and neutrophil influx as cigarette exposure increased from 1 to 2 (23). Similar results were observed with injection of CSC by Chujo et al. and Okamoto et al. where increasing CSC concentration increased the incidence and severity of CIA (28, 30). Notably, a plateau may exist where increased dosing of cigarette no longer increases arthritis severity, which was shown in both cigarette smoking models and CSC (23, 30).

In summary, when designing future experiments the genetics of the mice and timing and intensity of CS exposure should be considered as factors that modulate the outcome. However, the paucity of studies limits us in only questioning these as factors and more research should be directed to determine the effect of timing, intensity, and the interaction of these with genetics. Further, other factors that are known to enhance risk of RA such as sex and age are underresearched in these murine models and require future investigation. Understanding of these factors and how they modulate experimental arthritis and how this impacts response to therapeutics can lead to better personalized treatment strategies for patients with a given smoking history.

## Potential mechanisms

### Are the lungs the initial site of auto-antibody generation?

**Figure 1:**
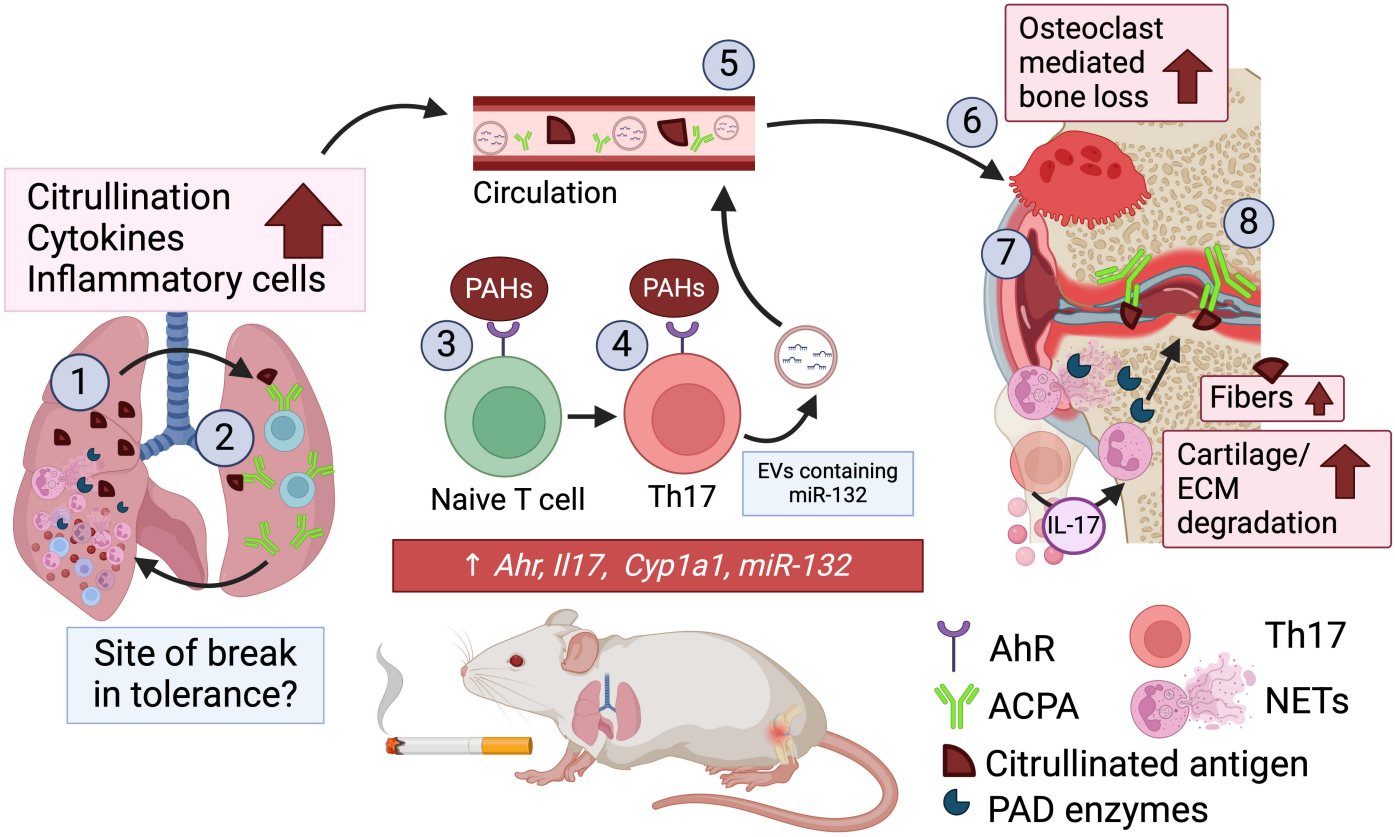
*Proposed mechanism of CS exacerbating experimental arthritis* (1). the first contact of CS is with the lungs which enhances inflammation and influx of immune cells in the lung, namely neutrophils which undergo NETosis to increase citrullination in the lung (2). The chronic inflammation and destruction of the lung may serve as the site of break in self-tolerance and generation of auto-reactive B cells against citrullinated proteins (ACPA) (3). The PAHs from cigarette smoke that enter the circulation from the lung engage the aryl hydrocarbon receptor (AHR) on T cells to drive a Th17 profile. Th17 cells are increased along with *Il17a* expression in lymph nodes and spleen which may then migrate to the joint (4). PAH interaction with AHR also drives increase in miR-132 production by Th17 cells which is packaged and secreted as extracellular vesicles (5). In the circulation there is increased EVs containing miR-132 from Th17 cells, as well as ACPA and citrullinated proteins, nicotine and its derivative cotinine, and heavy metals (6). The miR-132 drives osteoclastogenesis which drives bone damage (7). Increased ACPA in circulation binding to antigen in circulation and in the joint further enhances inflammation, increasing recruitment of inflammatory cells to the joint, such as neutrophils (8). Neutrophils undergo NETosis in the joint further driving citrullination within the joint providing increased antigen enhancing this loop of inflammation that drives bone and cartilage degradation.

By integrating findings from studies that demonstrated that CS exposure exacerbates CIA or AIA, we propose a potential mechanism by which CS exacerbates disease (**Figure 1**) (22–25). Inhaled CS first contacts the lung where resident cells respond by increasing inflammation and inflammatory cell influx into the lung in CIA and AIA mice (22, 25). Although the identity of the immune cells was not investigated by Kang or Heluaney et al, we hypothesize that these are neutrophils as others have shown that CS enhances neutrophil infiltration in the lungs of CS-exposed mice. The enhanced neutrophilia from CS possibly leading to increased NET formation, elevated local levels of PAD and citrullinated proteins (38, 39). The citrullinated proteins formed by NETs and ongoing activity of PAD enzymes may increase the pool of citrullinated auto-antigens in the lung, as shown by Kang et al. and others (22, 40, 41). The enhanced inflammatory milieu and increased burden of citrullination by CS creates the ideal environment for loss of self-tolerance against citrullinated proteins, especially for those with genetic risk, which is reflected by an increase in APCA in CIA mice exposed to CS (22).

### The impact of CS on the immune system, the role of Th17 cells

Several studies have investigated the impact of CS on the adaptive immune system, particularly the polarization of Th17 cells in driving disease. Clinically, Th17 cells are key immune mediators of RA, these cells and their cytokine IL-17 are enhanced in circulation, synovium, and synovial fluid (42). In CIA and AIA mice, CS enhanced Th17 cells through polycyclic aromatic hydrocarbons (PAHs) from CS engagement with the aryl hydrocarbon receptor (AHR) (23, 25). Consequently, the effect of CS on Th17 polarization was mitigated with AHR knockdown (23). Th17 expansion has been observed in RA and smoking appears to increase IL-17 in other airway diseases (43). Th17 cells may drive joint inflammation in multiple ways and cigarette smoke may influence their activity in murine arthritis. CS enhances the production of miR-132 by Th17 cells in AIA mice and RA patients, this microRNA is then transported by extracellular vesicles to promote osteoclast formation, the cell responsible for bone degradation and the formation of articular erosions (24). Th17 cells also recruit neutrophils to sites of inflammation, which is the most abundant cell type in synovial fluid of patients with RA. Similarily, CS exposure enhances neutrophils in joints of CIA and AIA mice (23, 44–46). Neutrophils may enhance RA through NET formation within the joint, which is exacerbated in AIA mice exposed to CS (25). As reviewed above, neutrophils release pro-inflammatory destructive cytokines and proteases that contributes to the degradation of the matrix in the joint (47–49). Further, PAD enzymes citrullinate the fibrous proteins in the joint, such as collagen, creating autoantigens for ACPA to bind to and drive inflammation (50). CS increased citrullination of the joints in CIA mice (22). The increased antigen in the joint then drives inflammation ultimately leading to increased pathology that is reflected by increased incidence and severity in response to CS (22–25). In summary, several aspects of the proposed mechanism must be confirmed, including whether burden of citrullination of the lung drives ACPA generation and if Th17 cells are enhanced in the joint to drive neutrophilia. Additionally, these mechanisms should be confirmed in humans allowing targeting of pathways enhanced in smokers contributing to exacerbated disease.

## Summary & future perspectives

In summary, the current literature on CS in murine models of inflammatory arthritis recapitulates many of the features of human RA, namely that CS exacerbates disease (22–25). CS enhances the key mediators of RA such as neutrophils, Th17 cells, and ACPA (2, 22, 23, 25). Deeper investigation of the mechanisms that lead to severe arthritis in smoking may lead to an enhanced understanding of the mucosal origins of RA. Additionally, the key factors outlined above which include genetics, timing and intensity of CS exposure should be further investigated to develop improved preclinical models that are most reflective of human disease. The CIA murine model offers the unique ability to investigate the effect of CS on the onset and progression of inflammatory arthritis, the development of autoantibodies and how CS exposures during or throughout specific stages of the pre-arthritis stage of CIA may alter outcomes. Finally, few studies have investigated the effect of smoking cessation on murine arthritis, which may provide insights in the utility of this strategy in individuals at-risk to develop RA. While the use of genetically modified animals can delineate the interaction between CS and genetics to enhance RA, proper use of controls can demonstrate an increased risk conferred by double risk factors rather than just one. Although the use of CSC has disadvantages, paired studies with CSC injection vs. inhalation represent an exciting avenue to explore how the lung interacts with CS to exacerbate RA and/or influence disease progression and severity.

Overall, various murine models of inflammatory arthritis represent the opportunity to investigate the effect of environmental factors, such as CS, on the development, progression, and response to therapeutics. This is an emerging field of investigation that has the potential to provide important insights to patients who smoke or have a history of smoking. Understanding important factors from preclinical data and how that impact outcomes will guide future investigations to better comprehend what should be considered when developing treatments for RA patients, and further enhance data that support smoking cessation.
